# Island mysteries in the spotlight: *Barbitistes kaltenbachi* and *Rhacocleis buchichii*, the only bush-cricket species endemic to Croatia (Orthoptera, Tettigoniidae)

**DOI:** 10.3897/zookeys.936.51599

**Published:** 2020-05-28

**Authors:** Rob Felix, Klaus-Gerhard Heller, Baudewijn Odé, Fran Rebrina, Josip Skejo

**Affiliations:** 1 IUCN/SSC Grasshopper Specialist Group, Gland, Switzerland; 2 Nijmegen, the Netherlands; 3 Grillenstieg 18, D-39120 Magdeburg, Germany; 4 University of Zagreb, Faculty of Science, Department of Biology, Division of Zoology, Animal Ecology and Zoogeography Lab,Rooseveltov trg 6, HR-10000 Zagreb, Croatia; 5 University of Zagreb, Faculty of Science, Department of Biology, Division of Zoology, Evolution Lab,Rooseveltov trg 6, HR-10000 Zagreb, Croatia; 6 Heinrich-Heine-Universität Düsseldorf, Institute of Molecular Evolution, D-40225, Düsseldorf, Germany; 7 De Bongerd 29, 6584 DG Molenhoek, the Netherlands

**Keywords:** Adriatic, Barbitistini, bioacoustics, Biokovo, Dalmatia, duet, ecology, Endangered, flightless, Hvar, IUCN Red List, Platycleidini, systematics, Vis Island, Vulnerable

## Abstract

Hvar Saw Bush-cricket *Barbitistes kaltenbachi* Harz, 1965 (Phaneropterinae: Barbitistini) and Lesina Bush-cricket *Rhacocleis buchichii* Brunner von Wattenwyl in Herman 1874 (Tettigoniinae: Platycleidini) are flightless orthopterans restricted to a narrow area in the Mediterranean part of Croatia, both originally described from Hvar Island. In this study, all available information on these two interesting species is presented: data on morphology, bioacoustics, distribution, habitat, and a key to identification of the species belonging to genera *Barbitistes* and *Rhacocleis* in Croatia. The songs of both *B. kaltenbachi* and *R. buchichii* are described here for the first time, with the former one being the second known example of a synchronising and presumably duetting species. Both species were reassessed according to the IUCN Red List criteria, where *B. kaltenbachi* should be considered an endangered species, while *R. buchichii* is suggested to be downgraded to a less threatened category. Biogeography and evolution of the species are briefly discussed.

## Introduction

Despite two centuries of research on Orthoptera in Croatia (Skejo et al. 2018), the only two bush-crickets endemic to the country, Hvar Saw Bush-cricket *Barbitistes kaltenbachi* Harz, 1965 and Lesina Bush-cricket *Rhacocleis buchichii* Brunner von Wattenwyl in Herman 1874, have escaped the eyes and ears of many researchers for quite some time. The knowledge of their distribution and biology is based merely on scattered documentation, with temporally and spatially well separated findings (Herman 1874; Harz 1965, 1969; Gausz 1970; Warchałowska-Śliwa et al. 2013; Wagner 2015; Puskás et al. 2018; Skejo et al. 2018).

Both species were originally described from the Island of Hvar (*Lesina* in Italian), one of Croatia’s Adriatic islands, belonging to the Central Dalmatian archipelago. Until now *Barbitistes kaltenbachi* has been regarded as a Hvar-endemic, while *Rhacocleis buchichii* has been found on other Dalmatian islands (Puskás et al. 2018) as well as in mainland Croatia (Gausz 1970, Wagner 2015, Skejo et al. 2018). The same is true for most other Orthoptera species that were originally regarded as Hvar-endemics, such as *Paramogoplistes novaki* (Krauss, 1888), *Chorthippus mollis lesinensis* (Krauss, 1888) and *Pholidoptera dalmatica maritima* Zeuner, 1931 (Willemse et al. 2009; Rebrina and Brigić 2017; Puskás et al. 2018; Skejo et al. 2018).

Since so little has been published about *Barbitistes kaltenbachi* and *Rhacocleis buchichii*, the authors of the current paper felt the need to present a comprehensive overview of the information available. This paper is written around RF’s finding of both species on the Island of Vis, well outside their formerly known distribution areas (briefly mentioned in Skejo et al. 2018). KGH’s records of *B. kaltenbachi* from two sites on Hvar Is. (briefly mentioned in Warchałowska-Śliwa et al. 2013; Rebrina 2014; Skejo et al. 2018) are presented in more detail, together with a comprehensive analysis of the bioacoustics of the species. Unpublished records of *R. buchichii* are presented, together with an overview of the published data. Distribution, habitat, ecology, and possible evolutionary patterns of *B. kaltenbachi* and *R. buchichii* are discussed. To facilitate recognition in the field, an identification key to the Croatian species of *Barbitistes* and *Rhacocleis* is provided.

## Materials and methods

### Study area

**Figure 1.**
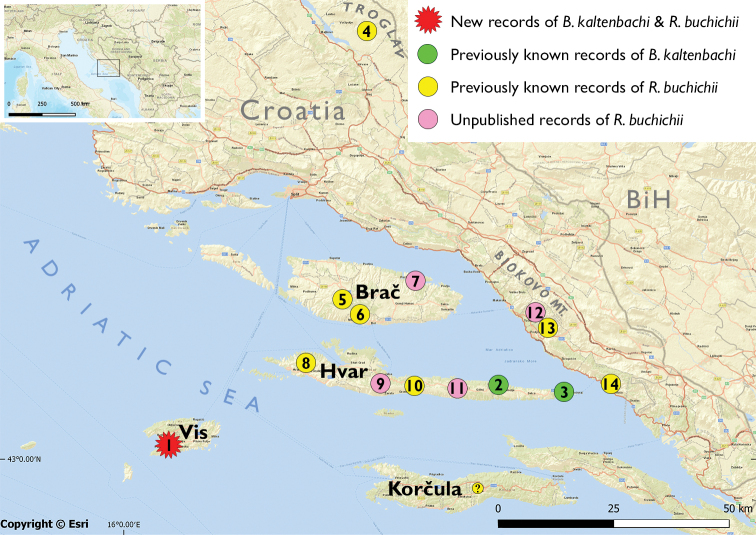
Distribution of the only known endemic bush-crickets (Tettigoniidae) of Croatia: *Barbitistes kaltenbachi* (localities **1**–**3**) and *Rhacocleis buchichii* (localities **1**, **4**–**14**, ?) **1** new records from Vis Is., Mt. Hum (present paper) **2** Hvar Is., Likova Glava, Bogomolje (present paper; Warchałowska-Śliwa et al. 2013; Rebrina 2014) **3** Hvar Is., Sućuraj (present paper; Rebrina 2014) **4** Velika Greda, Troglav Mt. (Skejo et al. 2018) **5** Brač Is., south of Nere-žišća (Werner 1919) **6** Brač Is., Vidova Gora Mt. above Bol (Puskás et al. 2018) **7** new record from Brač Is., valley near Pučišća (present paper, leg., coll. KGH, 31 May 2006) **8** Hvar Is., Brusje (Novak 1888; Ramme 1951) **9** new record form Hvar Is., Pitve and Vrisnik (leg. R. Kleukers, 13 Aug. 1996, NBC) **10** Hvar Is, Mt. Humac (Novak 1888) **11** new record Hvar Is., between Jelsa and Gdinj (present paper, leg., coll. KGH, 25 Jul. 1982) **12** new record from Biokovo Mt., above Tučepi (present paper, leg., coll. KGH, 28 May 2006) **13** Biokovo Mt. (Wagner 2015) **14** Zaostrog (Gausz 1970) "?" presence on Korčula Is. has never been confirmed (Harz 1969).

The Central Dalmatian islands (Fig. 1) form a tectonic unit composed of the geomorphologically similar islands of Vis, Hvar, and Brač (Borović et al. 1977). Unlike the typical Dalmatian orientation (direction NW to SE) exhibited by the majority of islands in the Adriatic Sea, these islands show *Hvar orientation* (direction W to E), witnessing their shared geological history. The Central Dalmatian islands harbour diverse habitats, e.g., rocky hills, small patches of forest, meadows (karst-poljes), ponds, and dry valleys. The backbone of the islands is formed by Mesozoic ridges of limestone and/or dolomite rock. The islands were connected to the Croatian and Italian mainland, by the ancient Po or Adriatic valley until approximately 11000–12000 years ago, when the sea level rose and the islands became separated (Lozić et al. 2012, Maselli et al. 2014). The Dalmatian coast is, just like the islands, warm and dry, with annual precipitation of only 700–900 mm. Isolated mountains (Svilaja Mt., Mosor Mt., and Biokovo Mt.) in the mainland have a mixed upper belt, rich in Mediterranean biota, with rare continental and oro-Mediterranean elements (Skejo et al. 2018).

### Identification and taxonomy

Specimens were identified using Harz (1969), the original species descriptions (Herman 1874; Harz 1965) and photographs of the type material on the Orthoptera Species File (Cigliano et al. 2019). Taxonomy follows the Orthoptera Species File (Cigliano et al. 2019).

### Institutional abbreviations

Specimens mentioned in this paper are deposited in the following collections:

**KGHC** Klaus-Gerhard Heller Collection

**MfN** Museum fur Naturkunde, Berlin, Germany

**NBC** Naturalis Biodiversity Center, Leiden, the Netherlands

**NHMUK** Natural History Museum, London, UK

**NMW** Naturhistorisches Museum Wien, Vienna, Austria

**RFPC** Rob Felix Collection, Nijmegen, the Netherlands

**ZSZJS** Natural History Museum Split: Josip Skejo Collection, Split, Croatia

### Measurements

Body length (from the frons to the tip of the abdomen), pronotum length in dorsal view, hind femora length, and ovipositor length (in females), were measured with a calliper of 0.1 mm precision.

### Analyses of *Barbitistes* bioacoustics

Male song (including the male-versus-male-interaction duets) was usually recorded in the evening, using a Sony WM-D3 cassette recorder and a SONY TCD-D7 DAT recorder with microphones Uher M 645 (Uher, Munich, Germany) and Sony ECM-121 (Sony, Tokyo, Japan). For the interactions (20 min duet recordings; 6 males) two males were placed separately, each in a plastic tube (Drosophila tube 28.5 × 95 mm, Biosigma, Cona (VE), Italy), standing side by side and each with a microphone placed inside (or on top of) the tube. Both microphones typically picked up both male sounds, but with quantifiable differences in amplitude. The output of each microphone was registered as a track of a stereo recording. After digitising the songs on a computer (sampling rate 44.1 kHz), oscillograms (after high pass filtering, typically around 1 kHz) and sound analyses were made using the following software: Turbolab (TL 4.0, Stemmer, Puchheim, Germany), Amadeus (Amadeus II, Martin Hairer, http://www.hairersoft.com), Audacity (Audacity 2.1.0; http://audacity.sourceforge.net) and Canary (Canary 1.2.4; Cornell Laboratory of Ornithology) on Apple. Due to the recorders’ restricted frequency response, data on frequency were not evaluated. Each data point for the time pattern is based on not less than 20 independent measurements (except series data), given as mean ± standard deviation (SD) and coefficient of variance (CV) (see Suppl. material 3).

### Analyses of *Rhacocleis* bioacoustics

Two sound recordings of two different individuals of this species have been made by Roy Kleukers (see Material examined under *Rhacocleis buchichii*), using a DCC-recorder (Philips DCC175) with a Shure Prologic condenser microphone. Sound recordings have been made in simple studio conditions at night, at 26–27 °C. Although the recordings are digital, some frequencies inaudible to humans were automatically removed before digitisation. Also, frequencies above ca. 20kHz are missing completely. The sound recordings are therefore not very useful for the analysis of frequencies. Yet, they are useful for temporal analysis of the song.

Temporal characters have been measured for both sound recordings using Wavelab 10 software (www.steinberg.net). A high pass filtering at 500Hz using phonetic software Praat 6.0.39 (www.praat.org) preceded the preparation of oscillograms for the Pitve specimen only.

Bioacoustic data of other *Rhacocleis* species has been derived from Ragge and Reynolds (1998)﻿, Heller (1988), and Massa et al. (2012) (analysis of the song of *R. japygia* by Paolo Fontana).

### Bioacoustics terminology

**Syllable**. sound produced during one cycle of wing movements. In *Barbitistes* only pulse-like closing hemi-syllables are known (Heller 1988) (Fig. 5A). In *Rhacocleis* both opening and closing hemi-syllables can be recognised; **trigger syllable**: pronounced syllable (*sensu*Stumpner and Meyer 2001); **syllable period**: time period measured from the beginning of a syllable to the beginning of the next; **echeme** (*sensu*Ragge and Reynolds 1998) or **chirp** (*sensu*Stumpner and Meyer 2001): a first-order assemblage of syllables; **verse**: a combination of echeme and trigger syllable; **verse period**: time period measured from the beginning of a trigger syllable to the beginning of the next (reciprocal value verse repetition rate). Details on songs of other *Barbitistes* and *Rhacocleis* species used for comparison can be found in Heller (1988), Ragge and Reynolds (1998) and Massa et al. (2012). For details on when and where *Barbitistes*-recordings were made see Suppl. material 3.

### IUCN Red List Assessment

Data from the previous IUCN assessments for *B. kaltenbachi* (Chobanov et al. 2016) and *R. buchichii* (Skejo 2014; Hochkirch 2016a) were used, together with the new data presented in this study to calculate new values of AOO (area of occupancy) and EOO (extent of occurrence) using GeoCAT Editor (Bachman et al. 2011) (available at http://geocat.kew.org). IUCN criteria were applied to suggest new Red List status qualifications for both species (IUCN 2001).

## Results

### Family Tettigoniidae Krauss, 1902

Subfamily Phaneropterinae Burmeister, 1838

Tribe Barbitistini Jacobson, 1905

#### Barbitistes kaltenbachi

Taxon classificationAnimaliaOrthopteraTettigoniidae

Harz, 1965

8058B0D5-55A8-51A3-8712-29B0758F22F9

http://lsid.speciesfile.org/urn:lsid:Orthoptera.speciesfile.org:TaxonName:10819

Figures 1 , 2 , 3 , 4 , 5 , 6 , 7 , 8

**Figure 2.**
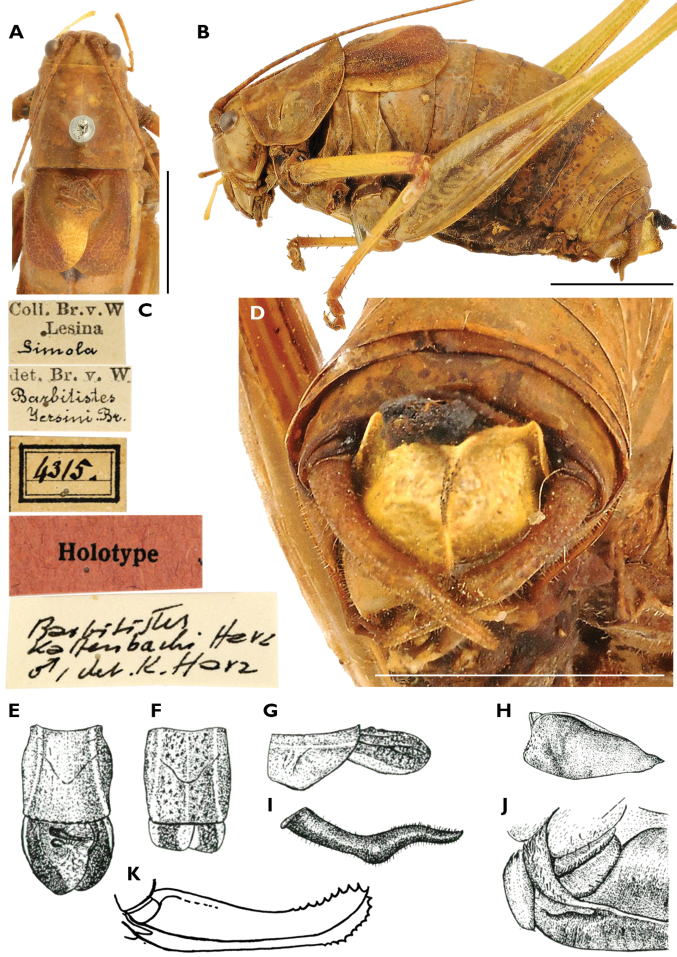
Photos of a syntype male of *Barbitistes kaltenbachi* by G. Puskás taken from OSF (Cigliano et al. 2019) (**A–D**) and drawings from Harz (1965, 1969) (**E–J**). The male specimen is labelled as a holotype, but since Harz (1965) did not designate a holotype, it should be labelled as syntype **A** pronotum and elytra of the syntype male in dorsal view **B** habitus of the male **C** labels of the male syntype **D** tip of abdomen with cerci and subgenital plate of the male **E** dorsal view of pronotum and elytra of a male **F** dorsal view of pronotum and elytra of a female **G** pronotum and elytrae of a male in lateral view **H** subgenital plate of a male seen from rear right **I** cercus of a male **J** base of ovipositor **K** ovipositor. Scale bars: 5 mm. Drawings are not to scale.

Barbitistes kaltenbachi Harz, 1965: Harz (1965): 443 (description of the species from Brunner von Wattenwyl’s collection, 12 ♂♂ syntypes, and 16 ♀♀ syntypes, depicted in details, measured, based on males and females from Hvar Is., and a female from Trieste) (see Fig. 2);
Barbitistes kaltenbachi Harz, 1965: Harz (1969): 75 (included in the key to species of the genus in Europe, depicted in details in figs 224, 237, 242–243, described and measured, cited from Hvar Is., and mentioned from a few mainland localities: Lukovo, Rijeka, Trieste);
Barbitistes kaltenbachi Harz, 1965: Galvagni and Fontana (1993): 204 (comparison with newly described B. vicetinus);
Barbitistes kaltenbachi Harz, 1965: Heller et al. (1998): 25 (listed in the checklist of European Orthoptera);
Barbitistes kaltenbachi Harz, 1965: Massa et al. (2012): 521 (presence in Italy, in the region of Trieste, unlikely);
Barbitistes kaltenbachi Harz, 1965: Warchałowska-Śliwa et al. (2013): 668–669, 671 (reported from Hvar Is., Bogomolje, number and shape of chromosomes determined, as well as sex determination system X0);
Barbitistes kaltenbachi Harz, 1965: Rebrina (2014): 4, 10–11, 14–16, 19, map Karta 1, Photo 1 (distribution in Croatia, Hvar cited as the only confirmed locality, new data from Bogomolje and Sućuraj, E corner of the island of Hvar, presence in Lukovo and Rijeka unlikely, Heller’s findings are the first since the description);
Barbitistes kaltenbachi Harz, 1965: Hollier and Bruckner (2015): 192 (Reported that type series should consist of 12 ♂♂ syntypes and 16 ♀♀ syntypes labelled ‘Insel Lesina’ and no specimens are deposited in Natural History Museum in Geneva. Since Harz did not designate a holotype all of the specimens in type series should be considered syntypes. Orthoptera collection in Vienna harbours 31 specimens identifiable as possible syntypes, of which one has been labelled as the holotype, one as allotype, and 16 as paratypes);
Barbitistes kaltenbachi : Chobanov et al. (2016) (assessed as Near Threatened in IUCN Red List);
Barbitistes kaltenbachi : Hochkirch et al. (2016b): 67 (listed among Near Threatened Orthoptera of Europe);
Barbitistes kaltenbachi Harz, 1965: Skejo et al. (2018): 19, 21 (endemic to Croatia, distribution in the country presented, as well as Rob Felix’s photograph from Hum, Vis Is.).

##### Type material.

**Figure 3.**
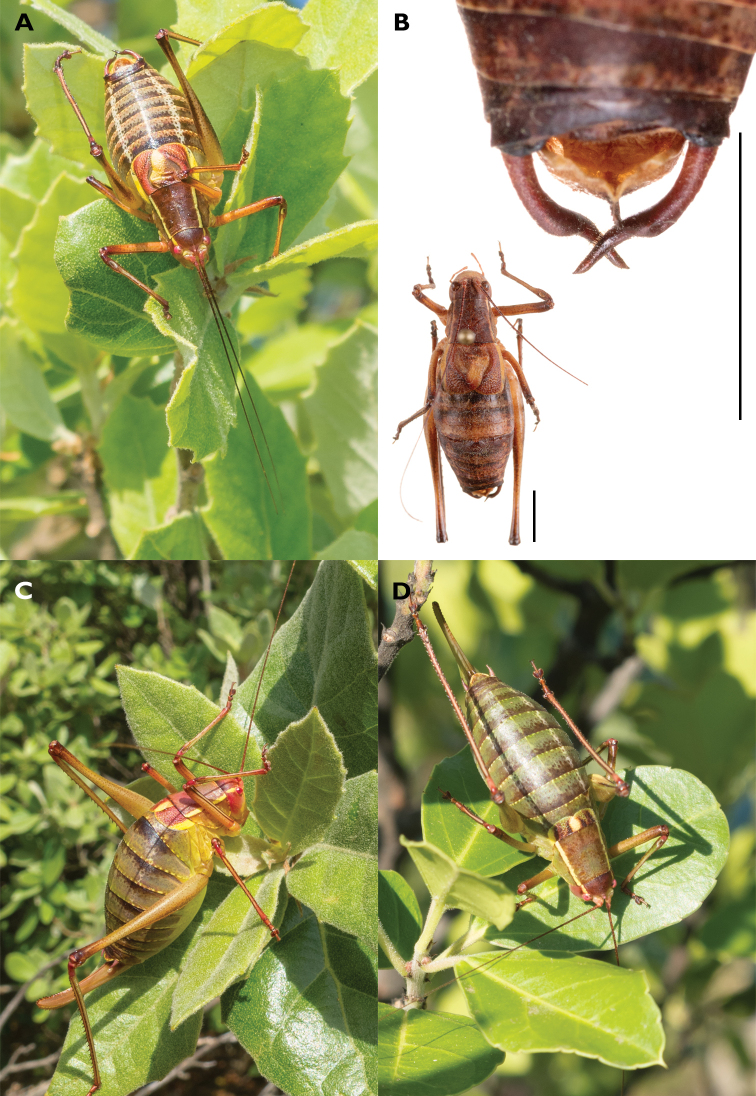
Habitus of *Barbitistes kaltenbachi*, Croatia, Vis Is., Mount Hum, 21 Jul. 2011 **A** male **B** collected male specimen and its cerci **C, D** females (photographs R. Felix). Scale bars: 5 mm.

Type specimens of *B. kaltenbachi* are deposited in NMW, Vienna. Harz’s (1965) description is based on 31 syntypes: 12 males (10 from Lesina (*Italian* for Hvar), 2 from an unknown locality, probably “Dalmatia”), 16 females (12 from Hvar, 1 from Trieste (Italy), 3 with unknown locality) and 3 nymphs from Lesina. A male syntype is erroneously labelled as holotype (Fig. 3).

##### Material examined.

Croatia • 2♂, 1♀; Vis Is., Mount Hum, Crikvica Sv. Duha; alt. 545 m a.s.l.; 43°02.13'N, 16°06.92'E; 21 Jul. 2011; R. Felix leg.; RFPC • 4♂, 2♀; Hvar Is., near Bogomolje, Likova Glava; 30 May 2006; M. & K.-G. Heller leg.; KGHC CH6738, CH6739, CH6740, CH6751, CH6752, CH6754 • 1♂; same data as for preceding; NBC CH6735 • 3♂, 1♀; Hvar Is., Sucuraj; 30 May 2006; M. & K.-G. Heller leg.; KGHC CH6736, CH6737, CH6755, CH6759.

##### New record on Vis Island.

**Figure 4.**
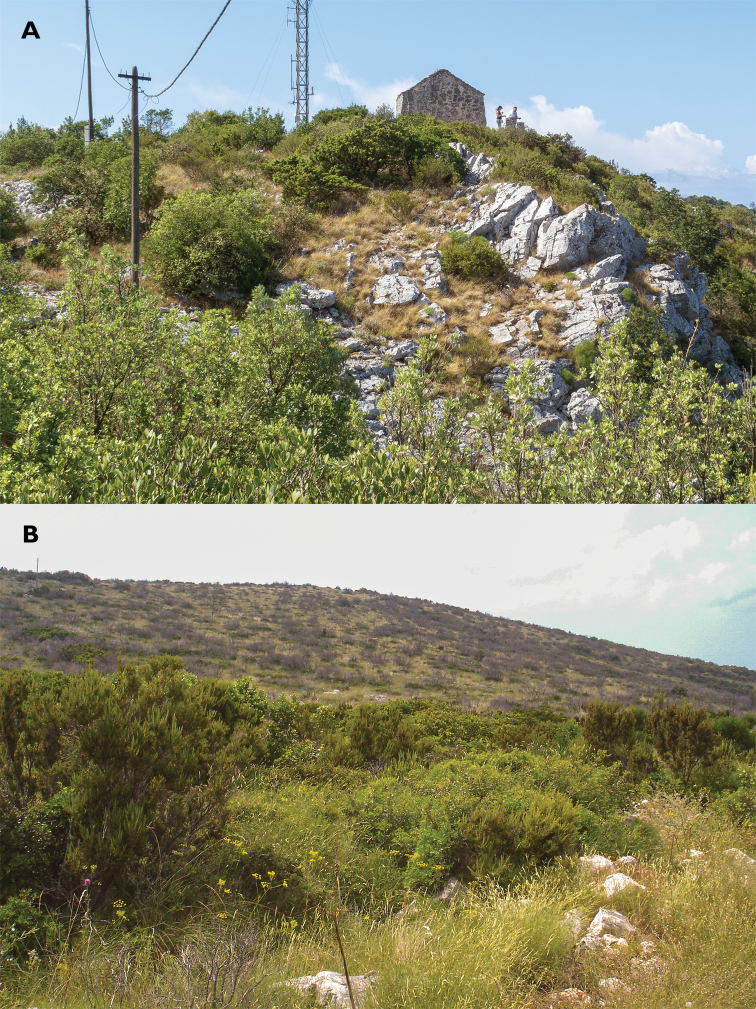
Collecting sites on Vis Is. and Hvar Is., Croatia **A** Vis Is., Mount Hum, St. Spirit Chapel (21 Jul. 2011). This locality is inhabited by both *Barbitistes kaltenbachi* and *Rhacocleis buchichii*. The vegetation is rich in Holm Oak bushes (*Quercus ilex*) (photograph R. Felix) **B** Hvar Is., Likova Glava, Bogomolje (30 May 2006), the habitat of *B. kaltenbachi* (photograph K.-G. Heller).

Hvar Saw Bush-cricket was found on 20 and 21 July 2011, near the Chapel of St. Spirit (Crikvica Sv. Duha) at Mount Hum, in the southwestern corner of Vis (43°02.13'N, 16°06.92'E, 545 m a.s.l.) (Figs 1, 3, 4A). Hum is the highest mountain on the island, with a peak reaching 587 m a.s.l. The observed specimens represent the first record of *B. kaltenbachi* on the Island of Vis, and outside of Hvar Is. Four individuals were observed, of which three were collected (see Material examined). Individuals were observed basking in the morning sun on outer leaves of Holm Oak (*Quercus ilex*), at a height of ca. 1.5 meters. *Barbitistes* was accompanied by Long-tailed Speckled Bush-cricket, *Leptophyes laticauda* (Frivaldszky, 1868) and Schmidt’s Marbled Bush-cricket, *Eupholidoptera schmidti* (Fieber, 1861) in the same habitat. The collecting site was inspected for only an hour, and a more comprehensive survey would probably have yielded more sightings.

##### Additional information on morphology.

The right tegmen of the female has several rows of stridulatory pegs, in resting position covered by the upper left tegmen. To produce sound, the pegs are probably contacted by the slightly enhanced inner edge or a sclerotised vein on the lower side of the left tegmen.

##### Distribution.

Harz (1965) originally described *B. kaltenbachi* from Hvar Is., Trieste (mainland Italy), and an unspecified locality (see Type material). Later, Harz mentions the species from Rijeka and Lukovo in mainland Croatia (Harz 1969). Massa et al. (2012) point out that the occurrence of *B. kaltenbachi* in mainland Italy (Trieste) is based solely on a female specimen. Since female specimens of *Barbitistes* are very hard to identify to species level, because of a high degree of overlap in interspecific characteristics (Nadig 1987), the record from Trieste is considered unreliable. Furthermore, Harz’s records from mainland Croatia are assumed to be based on females and are therefore regarded as doubtful. Therefore, we consider *B. kaltenbachi* as being absent from mainland Croatia and endemic to the Dalmatian Islands.

All known records of *B. kaltenbachi* are shown on the map in Fig. 1 and presented in Table 1. The first records of *B. kaltenbachi* after its description by Harz (1965, 1969) were the ones by KGH in 2006, on Likova Glava, near Bogomolje, and near Sućuraj, both on Hvar Is. (Fig. 1, 4B). In Warchałowska-Śliwa et al. (2013) and Rebrina (2014) coordinates are provided but after mapping, those appear to be incorrect, and are therefore omitted from Table 1. The distribution on both Hvar Is. and Vis Is. is probably much wider and the presence of the species on nearby islands such as Brač Is., Korčula Is., as well as neighbouring islets, is also possible.

**Table 1.** Known records of *Barbitistes kaltenbachi* with data on the sites and collection events. The #map correspond with the numbers in Fig. 1. Type specimens collected by various persons in different events (Harz, 1965) on Lesina (Hvar) are not included in the table, nor are depicted on the map in Fig. 1.

# map	Specimens	Location	Date of collection	Coll.	Reference
1	2♂, 2♀	Vis Is., Mt. Hum, Crikvica Sv. Duha, 43°02.13'N, 16°06.92'E, 545 m a.s.l.	20, 21 Jul. 2011	RFPC	This study
2	5♂, 2♀	Hvar Is., Bogomolje, Likova Glava	30 May 2006	KGHC	Warchałowska-Śliwa et al. (2013), Rebrina (2014)
3	3♂, 1♀	Hvar Is., near Sućuraj	30 May 2006	KGHC	Rebrina (2014)

##### Habitat.

No published data on the habitat of *B. kaltenbachi* are available. On Vis Is. the species has been found on bushes and small trees of Holm Oak, in maquis intercepted with scattered patches of grassy vegetation and bare limestone rock (Fig. 4A). The habitat on Hvar Is. is similar to that on Vis Is. but lacks rocky outcrops (Fig. 4B). *Barbitistes* is an arbusticolous genus, as its members are usually living in bushes, shrubs and on low trees (Galvagni and Fontana 1993; Pavićević et al. 2014). Thus, Hvar Saw Bush-cricket inhabits typical *Barbitistes*-habitat.

##### Song description.

**Figure 5.**
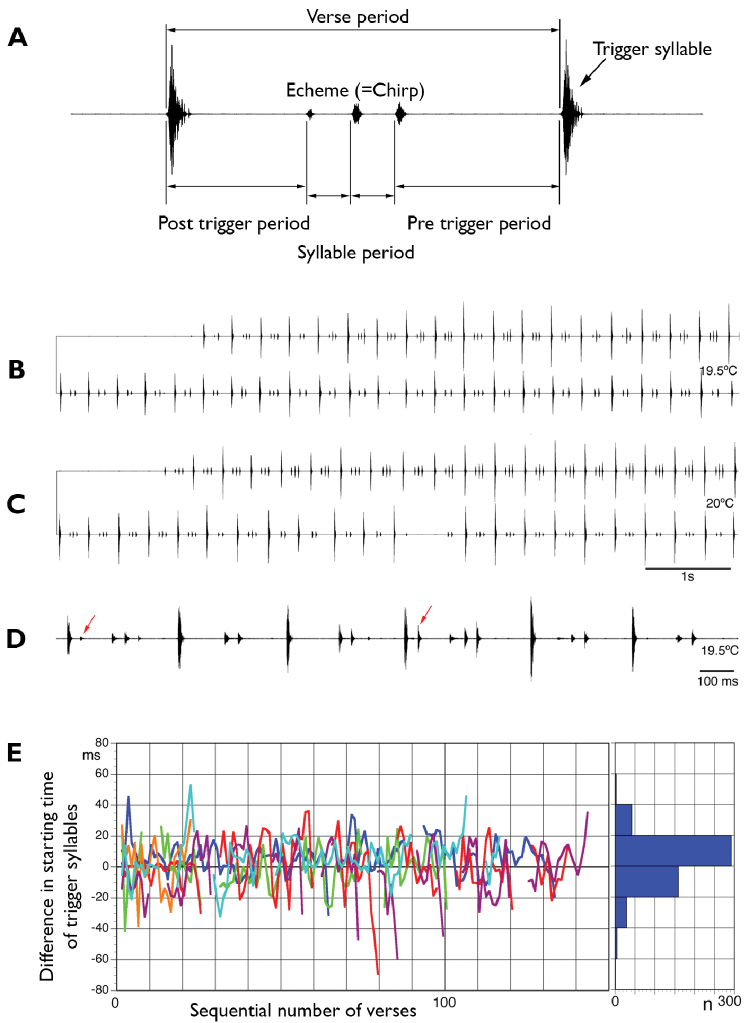
Bioacoustic data of *Barbitistes kaltenbachi***A** oscillogram showing bioacoustics terminology used in our study **B, C** details of the calling song (sections of 8 seconds) **B** second male starting in synchrony **C** second male starting anti-phasic, first male switching to synchrony **D** male song with extra syllables (red arrows, see text) **E** difference in starting time between the trigger syllables of two duetting males, always referring to a focal male; different colours indicate different male combinations; intervals in the lines mark duet pauses.

The male calling song consists of short (up to ca. 10 ms), pulse-like syllables without clearly recognisable impulses (tooth impacts). Syllables are arranged in a stereotyped pattern. One loud syllable, the *trigger syllable*, assumed to be the marker for female response (Stumpner and Meyer 2001), is followed by a group of two or three (rarely one or four) ‘softer’ syllables (echeme) (Fig. 5A). The trigger syllables are 6 to 16 dB louder than syllables within the echeme (Suppl. material 3: Table S3.1). After a short interval, the same pattern (verse) is repeated again and again (Fig. 5B). Periods range from 280–400 ms for the verse (verse repetition rate ca. 3 Hz), 120–160 ms for the post- and pre-trigger periods, to 40–50 ms for the syllables in the echeme (Suppl. material 3: Table S3.2). These sequences of verses are variable in duration but may last more than 30 s (Suppl. material 3: Table S3.3), at least while two males are in acoustic contact. During interactions males seem to prefer singing during the other male’s song pauses, but they are not inhibited. Songs overlap quite often, even for many seconds. In situations of overlap, males typically synchronise the pattern: they produce trigger syllables nearly at the same time (Fig. 5B, C). The delay between the trigger syllables of two synchronising males is typically below 20 ms (Fig. 5E) and the roles of leader and follower change often. After disturbances males are able to reach synchronicity within a few periods. Quite rarely, males add extra syllables (*sensu*Stumpner and Meyer 2001), ca. 50 ms after the trigger syllable (Fig. 5D). These song structures are known from several other *Barbitistes*-species and are interpreted as female response mimicking (Stumpner and Meyer 2001).

##### Species diagnosis.

**Figure 6.**
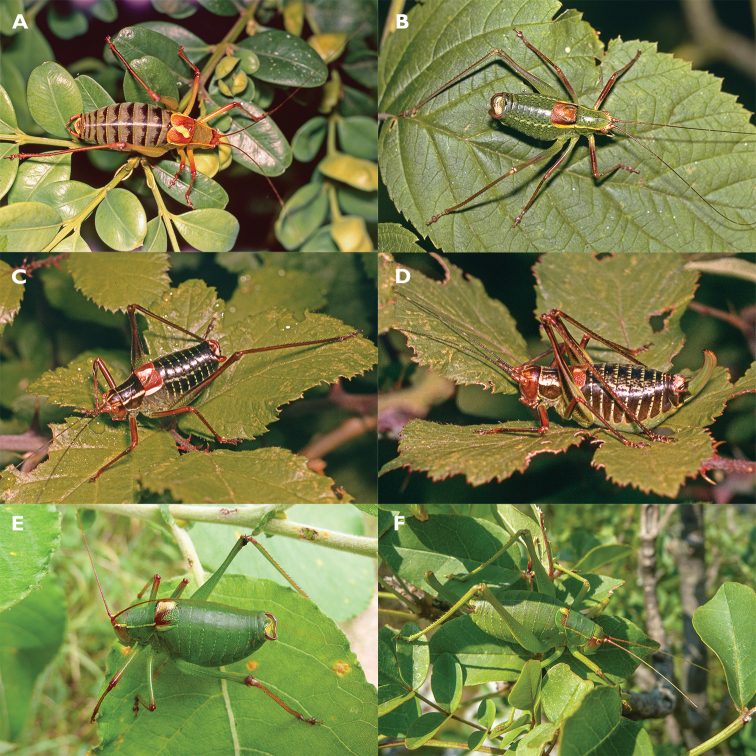
All *Barbitistes* species occurring in Croatia **A***B. kaltenbachi* (Croatia, Hvar, 30 May 2006) **B***B. serricauda* (Austria, Obir near Klagenfurt, 13 August 1980) **C** Male *B. ocskayi* (Montenegro, Lovćen pass above Kotor, 26 July 1982) **D** Female *B. ocskayi* (same locality as male) **E** Male *B. yersini* (Croatia, Grabovača, 7 Aug. 2018) **F** Female *B. yersini* (Croatia, Sniježnica, 20 Aug. 2018) (**A–D** photos K.-G. Heller **E, F** photos F. Rebrina).

**Figure 7.**
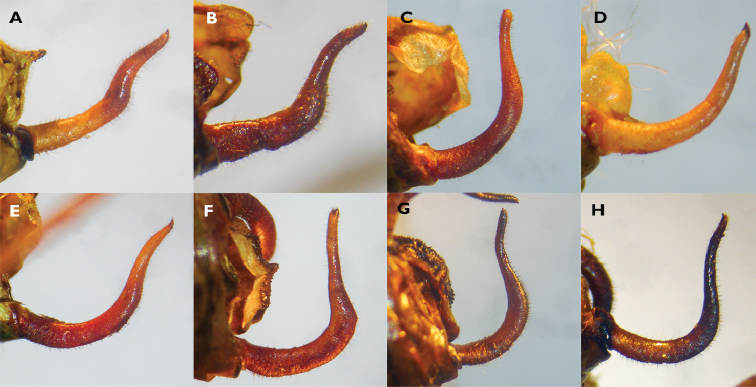
Male cerci of all members of the genus *Barbitistes***A–E**, **G, H** left cercus **F** right cercus flipped vertically **A***B. kaltenbachi***B***B. constrictus***C***B. alpinus***D***B. fischeri***E***B. serricauda***F***B. vicetinus***G***B. ocskayi***H***B. yersini* (photographs K.-G. Heller). Based on cercal morphology, *B. kaltenbachi* is most similar to *B. constrictus*.

Males of *Barbitistes kaltenbachi* can be distinguished from its Croatian congeners, *B. ocskayi* Charpentier in Ocskay et al., 1850, *B. serricauda* (Fabricius, 1794), and *B. yersini* Brunner von Wattenwyl, 1878 (Fig. 6), by the shape of the cerci (Fig. 7A). Male cerci in *B. kaltenbachi* are thickened in their mid-part. The proximal half of a cercus is in an abrupt but obtuse angle with the distal half, giving the cercus an angular sinuosity, like that of an open elbow. *Barbitistes constrictus* has nearly identically shaped cerci (Fig. 7B). All the other species of the genus have cerci of a different shape, in the absence of a thickened mid-part and in being more gradually curved, showing a smoother sinuosity in the apical part (Figs 7C–H), except for *B. vicetinus*, which has uniquely shaped cerci (Fig. 7F).

**Figure 8.**
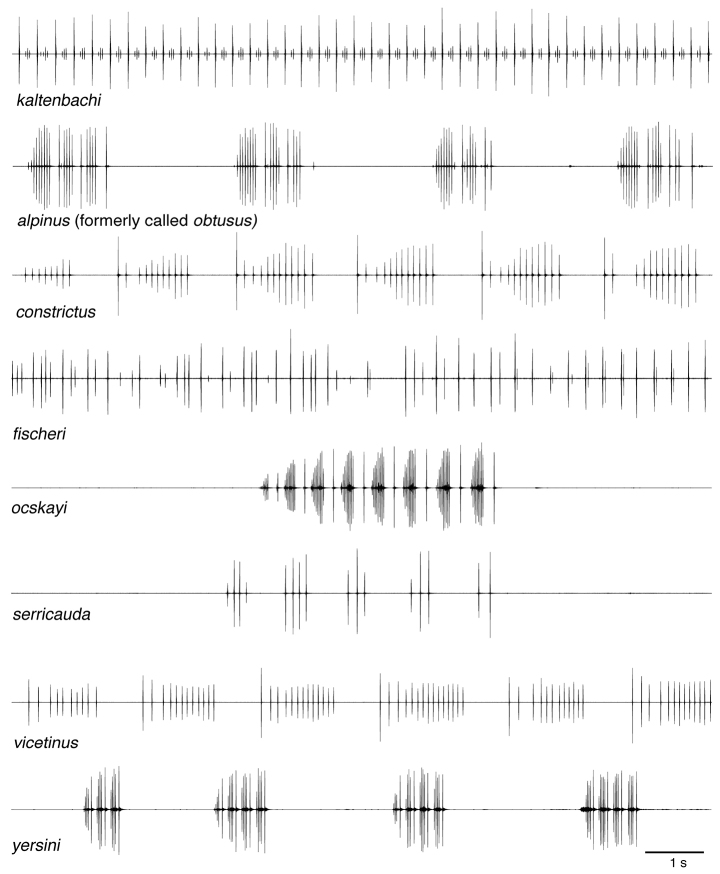
Oscillograms of male calling song (12 s sections) of all members of the genus *Barbitistes* (temperature range 21.5–25 °C).

The song of *B. kaltenbachi* differs distinctly from the songs of all other *Barbitistes*-species in the temporal pattern of the syllables (Fig. 8). Definite diagnostic differences between females cannot be given. Females of different species of *Barbitistes* show many similarities, some exhibiting intermediate characters (see e.g., Nadig 1987). In this paper, the photos of females are of specimens present together with males in the same bush and hence are identified as belonging to *B. kaltenbachi*. See Suppl. material 1: Table S1 for measurements of specimens in collections and literature data.

### Key to the species of the genus *Barbitistes* in Croatia (males only)

In Croatia, four species of *Barbitistes* occur, namely *Barbitistes kaltenbachi*, *B. ocskayi*, *B. serricauda*, and *B. yersini* (Rebrina 2014; Skejo et al. 2018). Males are easily identified to species level, but females are not. The key to males presented below is based on the morphology of cerci (angle and thickness) and subgenital plate (presence or absence of nose-like projection), and is adapted after Harz (1969). The key to the identification of females published by Harz (1969) has proven to be unreliable in practice, so it is not presented here.

**Table:** 

1	Cercus thickened in the middle, proximal half in an abrupt but obtuse angle with the distal part, giving the cercus an elbow-like appearance. Distal half angularly sinuous (Fig. 8) [currently known only from Hvar Is. and Vis Is.]	***Barbitistes kaltenbachi* Harz, 1965**
–	Cercus not thickened in the middle, but gradually narrowing from base to apex. Distal part slightly and smoothly sinuous	**2**
2	Subgenital plate with a nose-like projection, visible in lateral view. [Dubrovnik region; Istria, Kvarner with adjacent islands]	***Barbitistes ocskayi* Charpentier in Ocskay et al., 1850**
–	Subgenital plate without a nose-like projection, almost flat	**3**
3	Cercus halfway strongly incurved, with proximal and distal halves in an almost right angle (Fig. 8). Pronotum almost flat. Tegmina with a yellow triangle in the middle. [Common in the Dinaric Alps and Mediterranean Croatia]	***Barbitistes yersini* Brunner von Wattenwyl, 1878**
–	Cerci gradually curved (Fig. 8). Pronotum saddle-shaped. Tegmina dorsally uniformly reddish brown. [Pannonian Croatia; NW Dinaric Alps]	***Barbitistes serricauda* (Fabricius, 1794)**

### Reassessment IUCN Red List status of *Barbitistes kaltenbachi*

**Current status**. Near Threatened in Europe and EU28 (Chobanov et al. 2016).

**Area of occupancy** (AOO): calculated from the known data (ca.) 12 km^2^, maximal estimation 72–80 km^2^.

**Extent of occurrence** (EOO): calculated from the known data 90 km^2^, maximal estimation ca. 780 km^2^ (if all the hilly habitats in Hvar Is. and Vis Is. are included).

**Newly proposed status**. The species is reassessed here as Endangered. Up to now, the species has been known from only two Adriatic islands (Hvar and Vis). Inhabiting a restricted number of sites, it has an AOO of 12 km^2^ to maximally 80 km^2^, and a known EOO of 90 km^2^ (maximally estimated less than 800 km^2^). This makes it qualify for EN, following the criteria B1ab(iii)+B2ab(iii). Since there are only three sites at which this species occurs with certainty, only a few threat events may be enough to wipe out or threaten proportional parts of the population. Forest fires, for example, occur quite often on the Adriatic islands and can damage important parts of the species’ habitat, as can touristic and recreational developments and clear cutting of maquis and scrubland.

### Subfamily Tettigoniinae Krauss, 1902

Tribe Platycleidini Brunner von Wattenwyl, 1893

#### Rhacocleis buchichii

Taxon classificationAnimaliaOrthopteraTettigoniidae

Brunner von Wattenwyl in Herman 1874

726A34EF-2AF7-5651-8BC2-A9C3017CE1AC

http://lsid.speciesfile.org/urn:lsid:Orthoptera.speciesfile.org:TaxonName:2709

**Figure 9.**
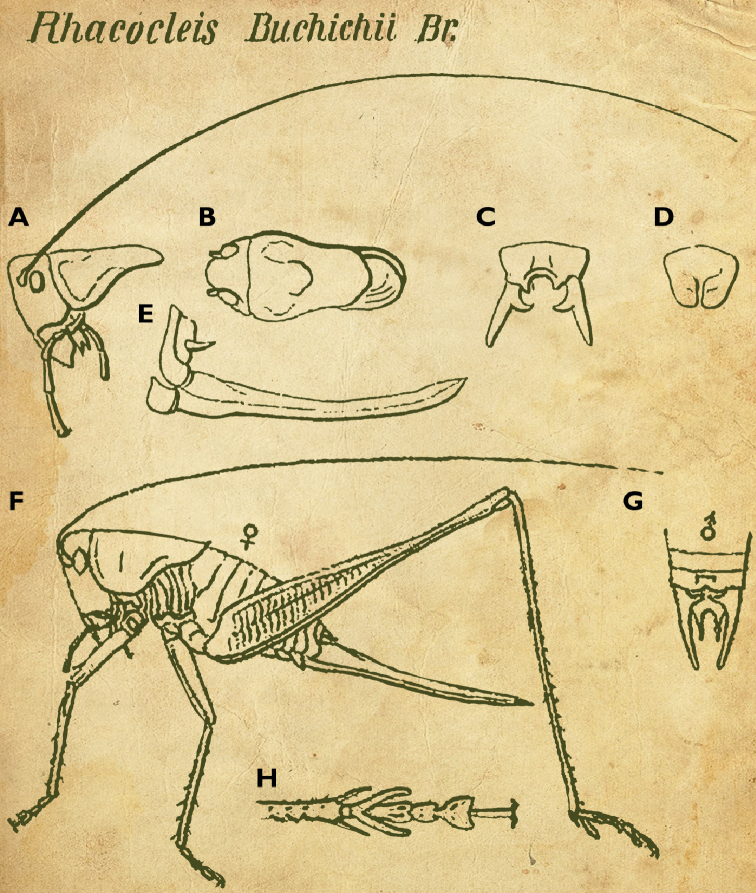
The first published spelling and the oldest drawings of *Rhacocleis buchichii* after Herman (1874) (**A–E**) and Brunner von Wattenwyl (1882) (**F–H**) **A** head and pronotum in lateral view **B** head and pronotum in dorsal view **C** cerci of a male **D** subgenital plate of a female **E** ovipositor **F** habitus of a female in lateral view **G** cerci of a male **H** hind leg details, apex of tibia and tarsus. Drawings not to scale.

**Figure 10.**
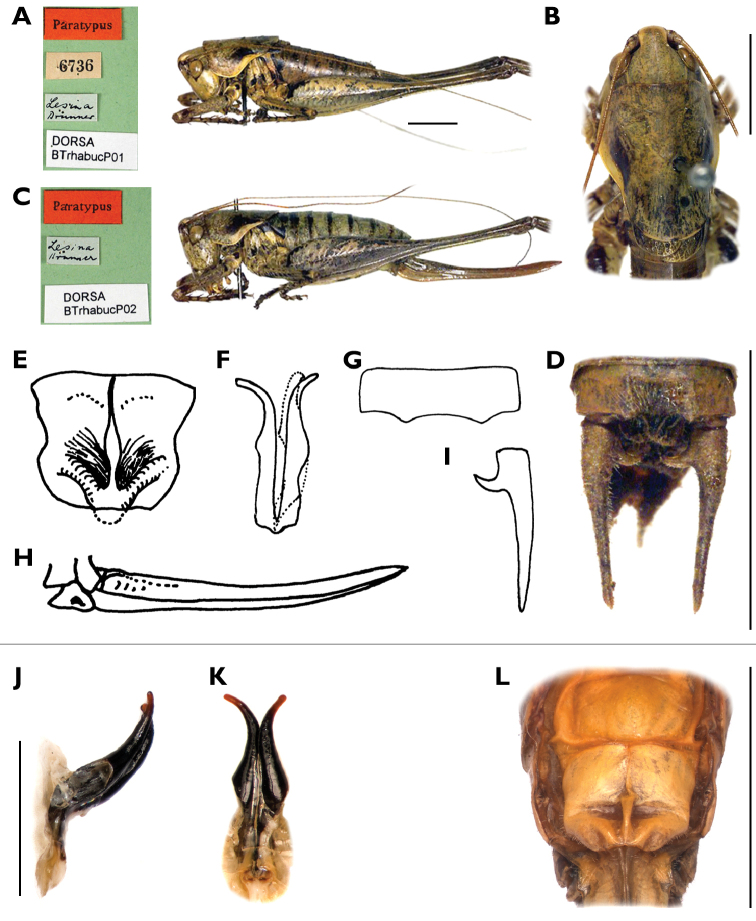
Photos of syntype male and female of *Rhacocleis buchichii* by Naskrecki, taken from OSF (Cigliano et al. 2019) (**A–D**) drawings from Harz (1969) (**E–I**) and details of specimens from Croatia, Hvar Is., near Pitve and Vrisnik, 13 Aug. 1996 (collection NBC) (**J–L**) (photos Luc Willemse, NBC) **A** male syntype and its label **B** pronotum and tegmina of the male in dorsal view **C** female syntype and its label **D** cerci and 10^th^ tergite of the male **E** subgenital plate of a female **F** titillators **G** 10^th^ tergite of a male **H** ovipositor **I** right cercus of a male **J** titillators lateral view **K** titillators caudal/posterior view **L** subgenital plate of a female. Scale bars: 5 mm (**A–D, L**); 1 mm (**J, K**). Drawings not to scale.

**Figure 11.**
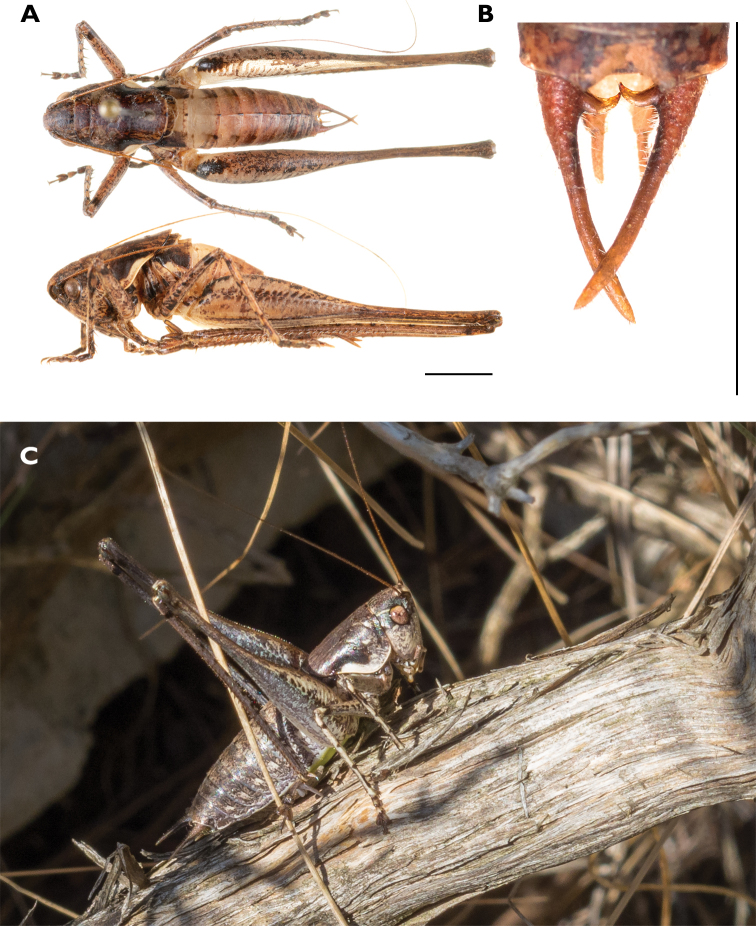
Habitus of *Rhacocleis buchichii*, Croatia, Vis Is., Mount Hum **A** male **B** cerci of the male **C** the same specimen in situ. Scale bars: 5 mm.

**Figure 12.**
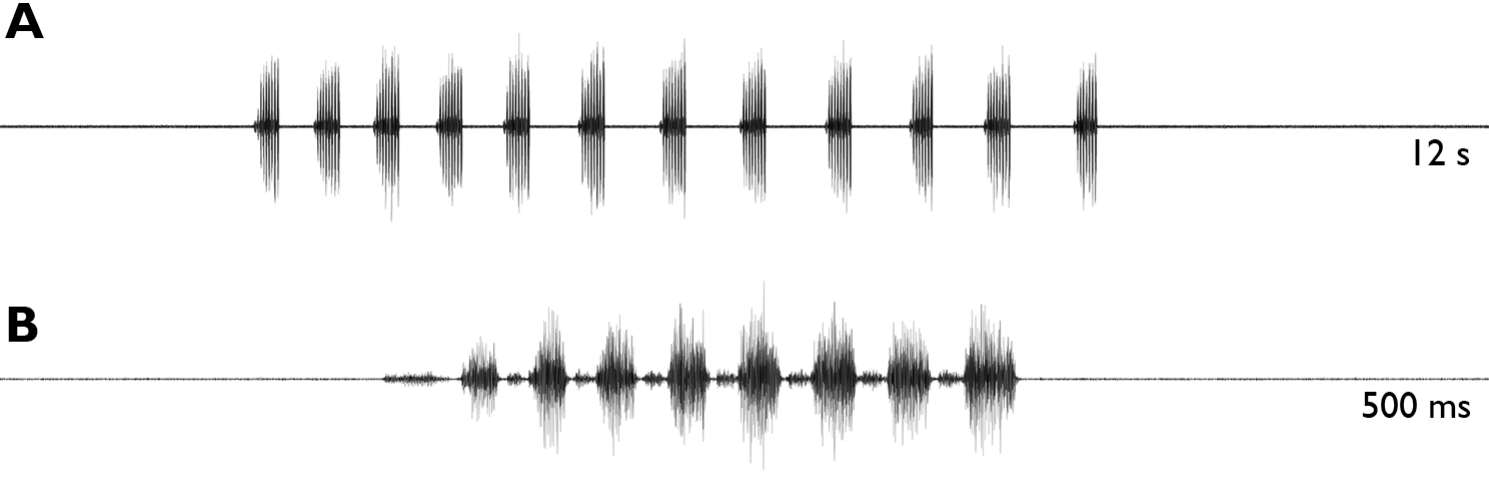
Bioacoustic data of *Rhacocleis buchichii***A** oscillogram showing one series of echemes (12 s) **B** oscillogram showing one echeme (500 ms).

Figures 1 , 9 , 10 , 11 , 12

Rhacocleis Buchichii Br.: Herman (1874): 201–202, plate III, figs 8–12 (original Brunner von Wattenwyl’s description accompanied with figures; erroneously calls buchichii type species of the genus) (see Figs 9, 10 in this paper);
Rhacocleis Bucchichii (O. Herman): Dubrony (1878): 21 (misspelled; reported the species from Liguria, based on misidentification of another Rhacocleis sp.);
Rhacocleis Bucchici Br.: Brunner von Wattenwyl (1882): 321 (misspelled; included in key), 322 (description, measurements, and Hvar, Lesina, designated as distribution);
Anterastes Bucchichi: Bucchich (1886): 382 (misspelled; Bucchich wrote a remark “In Luglio nei cespugli specialmente di erica; nei campi non si vede”, “In July found in bushes, especially of Erica; but not observed in fields” [= karst poljes]);
Rhacocleis Bucchici Br. 1874: Novak (1888): 129 (misspelled; provides first exact localities, Brusje and Humac on Hvar Is., and added data on Bucchic’s comments on species’ habitat: “Io ne ho trovato nel bosco ordinariamente fra il Cystus monspeliensis dal mese di giugno all’ ottobre, in agosto in casa introdotto forse colla frasca, negli orti fra i rosai, a Brusje e monte Humac in ottobre (25) sul Quercus”, “Usually I found them in bushes [or forest], under Cystus monspeliensis from June to October. In August in houses, probably with branches. Found in gardens under roses. In Brusje and Humac found on Quercus on October 25^th^”);
Rhacocleis bucchici Herman 1874: Redtenbacher (1900): 107 (misspelled; gives a short morphological description of the species with measurements and mentions the species to occur on the island of Lesina);
Rhacocleis bucchici Herm.: Jacobson and Bianki (1905): 400 (misspelled; colouration and morphology described in Russian, measurements provided, distribution cited ‘Dalmatia’);
Rhacocleis Buchichii , Herm.: Kirby (1906): 187 (listed in catalogue);
Rhacocleis Buchicii Br. 1874: Karny (1907a): 26 (misspelled; lists sp. in catalogue, citing Brunner von Wattenwyl, Novak and Redtenbacher);
Rhacocleis Bucchici : Karny (1907b): 131 (misspelled; mentions that the species has been known only from Hvar Is.);
Rhacocleis buchichii Herman: Caudell (1908): 5 (listed in the catalogue of Decticinae species, mentioned as endemic to Hvar Is.);
Rhacocleis bucchici Herm.: Werner (1919): 217 (misspelled; mentions species to occur on Brač Is., “south of Neresi, near the water tanks in the bushes, not common”. Also mentions “species until then only known from Lesina”);
Rhacocleis buchichii Herm.: Salfi (1924): 43, 45 (key to species);
Rhacocleis bucchichi Br.: Ramme (1951): 118 (reported to have found the species in hilly terrain under Pistacia terebinthus L. and noted that the species was timorous and difficult to catch);
Rhacocleis bucchichi Br.: La Greca (1959): 43, 148, fig. 115 (148) (misspelled; treats all the species of the genus and mentions buchichii to be present on Lesina Is., also shown on a distribution map of Italian species);
Rhacocleis bucchichi Hermann, 1874: Us (1967): 23 (misspelled; checklist of Yugoslavian Orthoptera, cited from Hvar, Brač and Korcula);
Rhacocleis bucchici Herm. 1874: Harz (1969): 429–441 (misspelled; included in key to European species, depicted in detail, figs 1343, 1361, 1396–1400, described and measured, distribution Hvar, Brač and probably also Korčula);
Rhacocleis buchichi Herm.: Gausz (1970): 131 (misspelled; first mainland record at Zaostrog along the Dalmatian coast);
Rhacocleis bucchicii Hermann, 1874: Heller (1988): 147, Abb. 146 F, I (F) (misspelled; mentions the species to occur on Hvar Is., lists a collecting event on 25 July 1982 and depicts the stridulatory file)
Pterolepis bucchicii (Herman, 1874): Heller et al. (1998): 37 (misspelled; species listed in checklist of European Orthoptera);
Rhacocleis bucchichi Herman, 1874: Willemse and Willemse (2005): 269 (misspelled; listed in checklist of Rhacocleis and Pterolepis species, 6 specimens from Dalmatia examined, in R. Kleukers and K.-G. Heller’s collections);
Rhacocleis buchichii : Skejo (2014) (assessed as Endangered species in IUCN Red list);
Rhacocleis buchichii Herman 1874: Wagner (2015): 37–41 (new record from Biokovo Mt., photographs provided);
Rhacocleis buchichii : Hochkirch et al. (2016a) (assessed as Endangered species in IUCN Red list, distribution Hvar, Brač and probably Korčula);
Rhacocleis buchichii : Hochkirch et al. (2016b): 20, 76 (listed among Endangered species in European Orthoptera fauna);
Rhacocleis buchichii Brunner von Wattenwyl in Herman, 1874: Skejo et al. (2018): 33 (author corrected to Brunner von Wattenwyl, species distribution in Croatia and Europe presented).

##### Historical misspellings.

Historically, the species name of *Rhacocleis buchichi* was misspelled a lot, for obvious reasons. It was even misspelled more often than cited correctly (see above). One specific misspelling, *bucchichi*, can be explained by the fact that the person after whom the species was named is Grgur/Gregorio Bučić/Bucchich (1829–1911), a Croatian naturalist with a surname that contains two consonants (voiceless postalveolar affricate consonant *č*, pronounced /tʂ/; and voiceless alveolo-palatal affricate ć, pronounced /tɕ/). These consonants were written in Croatian language in many different ways (ch, cch, ci, cci, chi) in the past, which has probably caused the confusion.

##### Type material.

Syntypes of *Rhacocleis buchichii*, one male and one female, are deposited in MfN, Berlin (Fig. 10). In the collection of NMW, Vienna, there are three specimens from the collection of Brunner von Wattenwyl (pers. comm. S Randolf). These three specimens are not labelled as types, but since they are part of the type-collection, they should be considered syntypes too.

##### Material examined.

Croatia • 1♂; Vis Is., Mount Hum; 43°02.13'N, 16°06.92'E; alt. 545 m a.s.l.; 21 Jul. 2011; R. Felix leg.; RFPC • 2♂; Hvar Is., between Jelsa and Gdinj; 25 Jul. 1982; K.-G. Heller leg.; KGHC CH0531 and CH2167 • 1♀; Biokovo Mt., above Tučepi; alt. 500 m a.s.l.; 43°16'N, 17°05'E 28 May 2006; M. & K.-G. Heller leg.; KGHC CH6779 • 1♂, nymph; Brač Is., near Pučišća; 31 May 2006, M. & K.-G. Heller leg.; KGHC CH6783 • 1♀; Troglav Mt., southern slope; alt. 850 m a.s.l.; 29 Aug. 2014; J. Skejo leg.; ZSZJS • 2♀; Hvar Is., 500 m south of Pitve; alt. 300 m a.s.l.; 13 Aug. 1996; R. Kleukers leg.; NBC RMNH.INS.960939 and RMNH.INS.1259083 • 1♂; Hvar Is., 500 m east of Vrisnik; alt. 150 m a.s.l.; 13 Aug. 1996; R. Kleukers leg.; NBC RMNH.INS.1259084; sound recorded • 1♂; Hvar Is., 500 m south of Pitve; alt. 300 m a.s.l.; 13 Aug. 1996; R. Kleukers leg.; sound recorded; specimen lost.

##### New record from Vis Island.

A single male individual of *Rhacocleis buchichii* was found under a *Juniperus* bush near the Chapel of St. Spirit (Crikvica Sv. Duha) at Mount Hum, in the southwestern corner of Vis Is. (43.036N, 16.116E, 545 m a.s.l.) (Figs 1, 2A, 11). Several photographs of the specimen were taken in its natural habitat (Fig. 11C), and it was subsequently collected (Fig. 11). As in the case of Hvar Saw Bush-cricket, this is the very first record of Lesina Bush-cricket for the island of Vis. It was recorded in the direct vicinity of the collecting site of *Barbitistes kaltenbachi*.

##### Distribution.

All known records of *R. buchichii* are depicted on the map in Fig. 1 and presented in Table 2. After its description from Hvar Is., the species has been found at scattered localities on the island and in a wide area outside the island: Zaostrog (Gausz 1970), Biokovo Mt. (present paper; Wagner 2015), Brač Is. (present paper; Puskás et al. 2018) and Troglav Mt. within the Dinara Massif (Skejo et al. 2018). Harz (1969) mentions the species’ occurrence on Korčula Is., but its presence on the island has never been confirmed.

**Table 2.** Known records of *Rhacocleis buchichii*, with data on the sites and collection events. Type specimens collected on ‘Lesina’ (Hvar Is.) and labelled as such are not included in the table, nor are depicted on the map in Fig. 1. Key: n/c, not collected.

Date of collection	map	Specimens	Location	Coll.	Reference
25 Oct. 1875	8	n/c?	Hvar Is., Brusje		Novak (1888)
25 Oct. 1875	10	n/c?	Hvar Is., Mt. Humac		Novak (1888)
20 Jul. 1912	5	n/c?	Brač Is., south of Nerežišća		Werner (1919)
18 – 25 Aug. 1939	8	7	Hvar Is., Brusje, below 500 m. a.s.l.		Ramme (1951)
Aug. 1964–1966	14	1	Zaostrog		Gausz (1970)
25 Jul. 1982	11	2♂	Hvar Is., between Jelsa and Gdinj	KGHC	
13 Aug. 1996	9	1♂ 2♀	Hvar Is., Pitve, 300 m a.s.l.	NBC	
13 Aug. 1996	9	1♂	Hvar Is., Vrisnik, 150 m a.s.l.	NBC	
18 Aug. 2002	6	1♂	Brač Is., Bol, Mt. Vidova Gora, 43°16.77'N, 16°37.14'E, 770 m a.s.l.		Puskás et al. (2018)
28 May 2006	12	1♀	Biokovo Mt., above Tučepi, 43°16'N, 17°5'E, 500 m a.s.l.	KGHC	
31 May 2006	7	1♂ juv	Brač Is., near Pučišća	KGHC	
21 Jul. 2011	1	1♂	Vis Is., Mt. Hum, Crikvica Sv. Duha, 43°02.13'N, 16°06.92'E, 545 m a.s.l.	RFPC	
29 Aug. 2014	4	1♀	Southern slope of Mt. Troglav, Greda, 43°49.33'N, 16°38.48'E, 850 m a.s.l.	ZSZJS	Skejo et al. (2018)
13 Oct. 2015	13	10♂♀	Biokovo Mt., 43°15.59'N, 17°05.57'E, 650 m a.s.l.		Wagner (2015)
16 Oct. 2015	13	5♂♀	Biokovo Mt., 43°15.32'N, 17°06.02'E, 650 m a.s.l.		Wagner (2015)

##### Habitat.

The first information on the habitat of *R. buchichii* was given by the name bearer himself; Bucchich (1886) found the species in July in a hilly terrain in bushes, especially *Erica*. He mentions that the species does not occur in fields (see Bibliography). Novak (1888) also mentions hilly areas as the prime habitat, adding that the species can be found from June to October under *Cistus monspeliensis*. Some individuals entered the house, probably as stowaways in collected firewood. In gardens, it was found under roses. Ramme (1951) reported the species to live under *Pistacia terebinthus* and noted that it was timorous and difficult to catch. Gausz (1970) found a specimen in the littoral zone near Zaostrog, in the undergrowth (karst-steppe vegetation of 3–10 cm high) in a stand of *Ficus carica* and *Olea europaea*. Kleukers (pers. comm.) found *R. buchichii* in 1996 on a rocky slope with low bushes around the villages of Pitve and Vrisnik, Hvar Is. Wagner (2015) found *R. buchichii* on Biokovo Mt., under Black Pine, *Pinus nigra*, while JS found it on Troglav Mt. under *Pistacia* sp. On 25 Jul. 1982, KGH found many specimens at night, walking and jumping on the road between Jelsa and Gdinj.

##### Song description.

We regard this song description as preliminary, as we were able to analyse the sound recordings of only two males. The calling song of *R. buchichii* consists of echemes that are repeated in a series of 4–13. However, in one of the recordings only the series of 2–5 occur. Echemes are repeated at a rate of ca. one or two echemes/s. In the available recordings, no continuous repetition of echemes is found. Echemes seem to have a more or less fixed structure, last ca. 160–220 ms and contain 7–9 syllables. Syllables are repeated at a rate of 40–50/s (26–27 °C).

##### Species diagnosis.

Within Tettigoniidae, the members of the Platycleidini tribe have either an unarmed prosternum or the prosternum bears two spines (Massa et al. 2012). The *Rhacocleis* genus belongs to the latter group, together with *Pterolepis* Rambur, 1838, *Antaxius* Brunner von Wattenwyl, 1882, *Anterastes* Brunner von Wattenwyl and *Yersinella* Ramme, 1933, among others.

Based on the shape of the cerci of the male and the subgenital plate of the female, La Greca (1959) placed *R. buchichii* into the *Rhacocleis neglecta*-species group (La Greca 1959), composed of *R. neglecta* (Costa, 1863), *R. japygia* La Greca, 1959 (both from central and southern Italy) and *R. buchichii* (Croatia). These species are characterised by 1) slender cerci with a decurved inner tooth close to the base (♂♂) and 2) a quadrate subgenital plate with a central keel and lateral depressions (♀♀). *Rhacocleis buchichii* is characterised by very small, *neglecta*-type titillators (Fig. 10J, K), but with a blunt and rounded apex (La Greca 1959). Cerci of the male are conical, delicate, very long and have a medial tooth protruding ca. 1/5 from their base (Figs 10, 11). La Greca (1959) provides a detailed description of the subgenital plate of the female: it presents a median carina and two lateral carinulas arranged obliquely and converging to the centre, towards the apex of the median carina. Transversal grooves on both sides of the median carina are situated more towards the apex of the subgenital plate than towards its base, and are limited posteriorly by the two lateral carinulas (Fig. 10L).

*Rhacocleis buchichii* is easily distinguished from its only congener in Croatia, *R. germanica* (Herrich-Schäffer, 1840). The cerci of *R. buchichii* males are very slender, while the males of *R. germanica* have more robust cerci, with a less elongated distal part. Each cercus of male *R. germanica* has a long and straight inner tooth. Females of *R. buchichii* have a rectangular subgenital plate with a median keel, which is armed at the tip (Fig. 10L), while *R. germanica* females have a prolonged, oval subgenital plate with an apical incision. The two Croatian species also differ in colouration. *Rhacocleis germanica* is more reddish and brownish tinted, without a white band on the paranota of the pronotum. *Rhacocleis buchichii* has darker and greyer tints and has a clear pale paranotal band (Figs 10, 11). See Suppl. material 2: Table S2 for the measurements of specimens in collections and literature data.

The song (Table 3) is different from the song of *R. germanica* in the number of syllables per echeme, being ca. ten in *R. germanica*, and 7–9 in *R. buchichii*. In *R. japygia* echemes are repeated in short series (2–8 echemes) or more or less continuously, with a repetition rate of ca. 1–2.5 echemes/s. Echemes consist of 5–7 syllables which are repeated at a rate of ca. 30/s. In *R. neglecta*, echemes are produced in long series or continuously, at a rate of 1–3 echemes/s. Echemes consist of 3–5 syllables. Syllables are repeated at a rate of ca. 25–40/s.

Echeme repetition rate and the number of syllables per echeme are considered to be the main distinguishing features of different species.

**Table 3.** Bioacoustic data of *R. buchichii*, *R. germanica*, *R. japygia*, and *R. neglecta*. Presented are echeme repetition rates per second, number of syllables per echeme, and syllable repetition rate per second. Data of *R. germanica* and *R. neglecta* obtained from Ragge and Reynolds (1998) and Heller (1988), data of *R. japygia* obtained from Massa et al. (2012).

Species	Echeme repetition rate (/s)	Syllables per echeme	Syllable repetition rate (/s)
*R. buchichii*	1–2	7–9	40–50
*R. germanica*	0,3–1	10	40
*R. japygia*	1–2,5	5–7	30
*R. neglecta*	1–3	3–5	25–40

### Key to the species of the genus *Rhacocleis* in Croatia

**Table:** 

1	Male cerci long and slender, inner tooth positioned at the basal fifth of the cercus, the apex of the tooth curved inward. Female subgenital plate rectangular, with a median keel thickened towards the apex. [currently known only from Brač Is., Hvar Is., Vis Is., and some inland and coastal mountains]	***Rhacocleis buchichii* Brunner von Wattenwyl in Herman, 1874**
–	Male cerci robust, inner tooth positioned just at the basis of the cercus, long and straight, in a right angle with the cercus. Female subgenital plate elongated, without a median keel and with an apical incision. [Common in the whole Mediterranean part of Croatia, including islands, and mountains; less common in Panno-nian region]	***Rhacocleis germanica* (Herrich-Schäffer, 1840)**

### Reassessment IUCN Red List status of *Rhacocleis buchichii*

**Current status.** Endangered (EN) in Europe and EU28 (Hochkirch et al. 2016a).

**Area of occupancy** (AOO): calculated from the known data (ca. 10 sites) 60 km^2^, maximal estimation 400 km^2^.

**Extent of occurrence** (EOO): calculated from the known data 3700 km^2^, maximal estimation ca. 7400 km^2^.

**Newly proposed status**. Lesina Bush-cricket inhabits the Adriatic islands of Hvar, Brač and Vis, as well as certain mountains in mainland Dalmatia. Since the species has recently been found on Vis Is. and its presence on Troglav Mt. has now been confirmed, its known range has extended significantly compared to the previous assessment. Based on the above calculations, we propose the species to be downgraded to a less threatened category.

The species occurs in Natura 2000 protected areas (Vis Is., Hvar Is., Brač Is.) and in a protected natural park (Biokovo Natural Park), but it is expected to occur outside the protected areas as well, where the main threats to the species’ survival persist. Based on numerous references, the habitat type of the species, to be considered in a future reassessment, has to be extended with scrubland (see above, under Habitat).

## Discussion

### Dalmatian endemics from a biogeographical perspective

The Adriatic islands are of a rather young evolutionary age (Malvić 2016) compared to large Mediterranean or Greek islands (Robertson and Dixon 1984, Willemse et al. 2018). Therefore, the rate of endemism on the Adriatic islands is relatively low (Kenyeres et al. 2009). During most of the time from Oligocene to Pleistocene, the Adriatic islands seem to have been a part of the mainland Balkans (Giuli et al. 1987). From the lower Pliocene, when the Adriatic sea was large and extended far into the north-west, through the Würm ice age (ca. 100,000–11,700 years ago), when the sea level was low, as well as in the early Holocene, when the sea level increased, the islands were connected to the mainland (Velić and Malvić 2011, Malvić 2016). They may have been isolated during some interglacials, but these periods were probably short.

Nevertheless, a few endemics known from the central Dalmatian islands, mainly documented in plants (Bogdanović et al. 2014), may justify the status of this island group as a distinct biogeographic unit. Among animals, however, very few examples are known, most of which refer to troglobitic taxa. Of particular note is the troglobitic beetle genus *Spelaeobates* Müller, 1901, endemic to the central Dalmatian islands and adjacent Croatian mainland. *S. kraussi* Müller, 1903 and *S. pharensis* Müller, 1901 are Brač and Hvar endemics, respectively (Pretner 1968, Gottstein Matočec et al. 2002) while *S. novaki* Müller, 1901 is found on Dugi otok Is. and adjacent Croatian mainland, at the foot of Velebit Mt. (Jalžić 1983). Allegrucci et al. (2017) recently mentioned a new, yet undescribed species of *Troglophilus* (T. sp. 1) from the caves on Mljet Is. However, these troglobitic and troglophilous forms live in special habitat ‘islands’, not necessarily related to true islands.

The terrestrial fauna of most Croatian islands still remains profoundly understudied and the Orthoptera of the Central Dalmatian islands, in particular, have never been studied systematically (Skejo et al. 2018). At the present state of knowledge, however, the occurrence of endemic or subendemic Orthoptera species is surprising and noteworthy. How and when could these forms speciate? Surprisingly, the available data indicate rather different histories for the two species studied in this paper.

To start with the less complex case, the Lesina Bush-cricket *Rhacocleis buchichii* is endemic to Dalmatian islands (Brač, Hvar, Vis) and mountains (e.g., Troglav and Biokovo), and occurs sympatrically with *R. germanica*, the latter being very common in the Mediterranean region of Croatia. The two species show clear morphological differences and do not seem to be closely related. On the other side of the Adriatic Sea, in Italy, two species presumably closely related to *R. buchichii* and each other occur, namely *R. neglecta* and *R. japygia* (La Greca 1959). *Rhacocleis neglecta* is widespread in the Italian mainland, while *R. japygia* occurs in a small area between Basilica and Puglia (Massa et al. 2012). All three species of the so-called *neglecta*-group (*neglecta*, *buchichii*, *japygia*; La Greca 1959) have similar cerci in males and quadrate subgenital plates in females. Thus, it is highly probable that these species share the most recent common ancestor. La Greca (1959) considers *R. buchichii* as originating from the Apennines, Italy. The ancestor of *R. buchichii* may have inhabited the area currently covered by the Adriatic Sea during one of the periods when the sea level was low (e.g., during Würm; Malvić 2016). After the sea level rose and the Balkan Peninsula became separated from the Italian Peninsula, speciation became possible.

Despite the fact that the similarity between the three species was mentioned as early as 60 years ago (La Greca 1959), a comprehensive comparative study has never been conducted. In terms of bioacoustics, the songs of the three allopatric *Rhacocleis* species are relatively similar, but all four mentioned species of this genus can be identified by their songs. However, very few sound recordings of these species exist and a more thorough study of their bioacoustics, including female behavioural response experiments, is required to test this hypothesis.

The situation with Hvar Saw Bush-cricket *Barbitistes kaltenbachi* is quite different and much more complex. This flightless bush-cricket is endemic to two Dalmatian islands (Hvar Is. and Vis Is.). The song of *B. kaltenbachi* is unique within the genus and does not resemble that of any other known species (Figs 5, 6). On Hvar Is., *B. kaltenbachi* occurs sympatrically with *B. yersini*, a common and widespread species in the Western Balkans (Hochkirch et al. 2016a, Skejo et al. 2018), with an isolated, trans-Adriatic occurrence in Central Italy (Massa et al. 2012). The true origin of this isolated population would be an interesting subject for a bioacoustic/genetic study.

According to the cercal morphology, *B. kaltenbachi* could be closely related to *B. constrictus*, their cerci being nearly identical (Fig. 8). On the other hand, *B. constrictus* has a completely different distribution and habitat. It inhabits central, northern and eastern Europe (Harz 1969) and is typically found in coniferous forests (Hochkirch et al. 2016b), whereas *B. kaltenbachi* is a species of dry and hot maquis.

Looking at the distribution ranges of other *Barbitistes* species, one can recognise an intriguing gap between the northern and southern distributional areas of *B. ocskayi* (see Hochkirch et al. 2016b, Skejo et al. 2018), in which the distribution of *B. kaltenbachi* seems to fit perfectly. If *B. kaltenbachi* were an offshoot of *B. ocskayi*, it would be relatively young (perhaps isolated during one of the interglacials), but in this case it means that its unique song and distinctive cercal morphology evolved very fast. Since both characters are known to be under sexual selection (Lehmann 1998), a rapid evolution seems possible. Under these premises, it would be particularly interesting to get a molecularly based estimate of the age of the species involved. If the distribution of *kaltenbachi* is restricted to Hvar Is. and Vis Is. – during a quick search we only found *B. yersini* on Brač Is. and the mainland opposite to Hvar Is. – this could be ascribed to its occurrence (or origin?) in only one of the two separate local Würm glacial refugia, that were situated south of the Neretva River (see Podnar et al. 2004).

Based on the shape of the subgenital plate, *B. kaltenbachi* is similar to *ocskayi*. Based on cercal morphology, however, *B.﻿ ocskayi* and *B.﻿ yersini*﻿ seem to resemble each other more. *Barbitistes vicetinus* is a species restricted to Northern Italy, which exhibits an isolated occurrence comparable to that of *B.﻿ kaltenbachi*﻿. Despite a superficial resemblance of the male cerci of *B.﻿ kaltenbachi*﻿ and *B.﻿ vicetinus* (the latter also has a somewhat thickened mid-part; Fig. 7F) the authors consider the cerci of the two species very different from each other. *Barbitistes vicetinus*’ cerci show a right angle between the proximal and distal parts, and almost lack any sinuousity in the distal part (compare *B. kaltenbachi* in Key to the species). Furthermore, their songs are different (Fig. 8).

### Acoustic behaviour of *Barbitistes kaltenbachi*

*Barbitistes kaltenbachi* belongs to Phaneropterinae, a bush-cricket subfamily in which females of most species do not respond to male songs only by phonotactic approach, but also react with their own acoustic signals. These sounds are used by males for locating females (see Heller et al. 2015 for a review). Female responses are often short, occur very fast after releasing male the song element (in less than 100 ms; see Heller et al. 2018) and are scarcely detectable by predators, but can be exploited by conspecific males (Villarreal and Gilbert 2014). Therefore, it is not surprising that a variety of song modifications which can be interpreted as forms of defense against eaves-dropping rivals, are known (Heller and Hemp 2017, Heller et al. 2017). The rivals, on the other hand, can attempt to mask female response (Bailey et al. 2006).

Male calling songs of all species belonging to Barbitistini*sensu stricto* (genera *Barbitistes*, *Metaplastes*, and *Ancistrura*) are characterised by short, isolated syllables showing a species-specific pattern (Heller 1988). They mostly contain a short sequence of syllables (up to ten) followed by a larger interval, after which an isolated syllable is produced. This syllable is called trigger syllable, because a female ready to mate responds directly to it. *Barbitistes* males (e.g., *B. serricauda* and *B. kaltenbachi*; Fig. 5) sometimes produce so-called “extra syllables” (Stumpner and Meyer 2001) at the time of an expected female response, possibly to hinder the rivals’ ability to locate answering females. However, there are almost no data describing the calling behaviour of two or several males singing together. A rival male could e.g., disturb the silent interval (before the next trigger syllable of another male) by sounds reducing the probability of female response. In some species, trigger periods are certainly long enough for such attempts (e.g., *Ancistrura nigrovittata*, *B. constrictus*, *B. serricauda*), but in others, sequences of verses are probably too short (e.g., *B. alpinus*, *B. yersini*), making the prediction of the trigger periods difficult. *Barbitistes kaltenbachi* songs are relatively long, so the rivals’ disruption attempts should not be difficult. However, a rival would arguably not gain much from such behaviour, because its own song could be disturbed by the song of the ‘attacked’ male in a similar way. Thus, it could be more worthwhile to synchronise the song as closely as possible with the song of a neighbour.

During the duets of *B. kaltenbachi*, trigger syllables/verses of both males started within only 20 ms (Fig. 5). Some duetting frogs have a mean difference of 79 ms (Legett et al. 2019). The shortest onset difference tested by Snedden and Greenfield (1998) in a synchronising tettigoniid was 26 ms. When synchrony is near-perfect (*sensu*Legett et al. 2019; delays of 5 ms or less), as observed here in many instances, males increase the amplitude of their signals by overlapping. In any case, both males are still able to hear female responses, provided the female is in the hearing range of both. This system works even if males use auditory time windows, as reported from other Barbitistini (Heller and von Helversen 1986). To our knowledge, synchronising in duetting bush-cricket species has been observed only in *Amblycorypha parvipennis* (Shaw et al. 1990). This species has syllables (called phonatomes in Shaw et al. 1990) with a duration of 100 ms. Female responses follow ca. 120 ms after the beginning of a male syllable. In Barbitistini, synchronising would have been considered unlikely due to the fast female response and narrow auditory time windows.

Unfortunately, as nothing is known about female acoustic behaviour or the potential male reaction, the hypotheses regarding *B. kaltenbachi* acoustic behaviour can only be based on what is known of other species. *Barbitistes* females respond to the trigger syllable after a delay of ca. 40 ms (Stumpner and Meyer 2001), probably caused by an audio-motoric reflex (named acousto-motorical reflex in *Ancistrura nigrovittata* by Dobler et al. 1994). In this case, a preference for the leading call would be obvious. Females would (and can only) respond to the first sound. This is comparable to a precedence effect discussed in the context of synchrony and alternation in chorusing animals (Greenfield 2002). However, males are not necessarily under pressure to call first. This depends a lot on the specific borders of the auditory time window. The trigger syllable of the leading male may start the auditory time window not only in the female, but also in the male follower. Ideally, it should not be reset by the follower’s own trigger syllable. On the other hand, singing at a different time from the leading male may result in covering the female response. Thus, exact synchronisation might be the optimal strategy. Of course, much more data, especially from females, are necessary to prove this hypothesis, but it seems a fascinating prospect for acoustic co-operation – even if it is considered to ‘make the best of a bad situation’.

### Concluding remarks

With this paper, we attempted to enhance the information on the distribution of two Croatian endemic bush-crickets, as well as the knowledge of their morphology and bioacoustics. The songs of *Barbitistes kaltenbachi* and *Rhacocleis buchichii* are described here for the first time. The IUCN Red List status has been reassessed here for both species; we suggest *R. buchichii* to be downgraded to a less threatened category, while *B. kaltenbachi* should be upgraded to ‘Endangered’.

The knowledge of the biology of both species is still scarce. *Barbitistes kaltenbachi* is suspected to be active early in the season, while *R. buchichii* is probably active late in the season, based on many records of this species from October (see Tables 1, 2). JS visited Hvar Is. two times, in late July and late August 2017, focusing on finding both species, but without success. Both dates were apparently too late in the season for *B. kaltenbachi*, taking into account that KGH collected specimens of this species on Hvar Is. at the end of May, without observing nymphs at that time.

The fact that the type series of *B. kaltenbachi* consists of a fairly large number of specimens (22) from the same locality (Hollier and Bruckner 2015) could suggest that the species exhibits gradations in certain years. Gradations are a well-known aspect of the biology of other *Barbitistes* species (Galvagni and Fontana 1993, Stumpner et al. 2015), some of which were, and still are, sometimes characterised as pest species (e.g., Bei-Bienko 1954, Cavaletto et al. 2018). However, outbreaks of *B. kaltenbachi* have never been reported.

To enrich the knowledge of the Orthoptera fauna of Vis Is. and other Adriatic islands, the authors would like to suggest visiting entomologists to pay attention to these and other species, and enter their sightings, accompanied by photos, on websites such as Observation (www.observation.org) or iNaturalist (www.iNaturalist.org).

## Supplementary Material

XML Treatment for Barbitistes kaltenbachi

XML Treatment for Rhacocleis buchichii

## Supplementary materials

Supplementary material 1 *Barbitistes kaltenbachi* morphometrics Data type Microsoft Word Document (.docx) Explanation note Table S1. Morphometrics of Hvar Saw Bush-cricket. Published measurements (Harz 1965, 1969) are compared with the measurements of specimens collected on Vis Is. and Hvar Is. Shown are body length (from the frons to the tip of abdomen), pronotum length (in dorsal view), length of the visible part of tegmina, hind femur, and ovipositor length (in females). All measurements are in mm. [* the tip of abdomen of the other male was eaten by a wasp during preparation] https://binary.pensoft.net/file/414989This dataset is made available under the Open Database License (http://opendatacommons.org/licenses/odbl/1.0/). The Open Database License (ODbL) is a license agreement intended to allow users to freely share, modify, and use this Dataset while maintaining this same freedom for others, provided that the original source and author(s) are credited.Rob Felix, Klaus-Gerhard Heller, Baudewijn Odé, Fran Rebrina, Josip Skejo

Supplementary material 2 *Rhacocleis buchichii* morphometrics Data type Microsoft Word Document (.docx) Explanation note Table S2. Morphometrics of Lesina Bush-cricket. Published measurements (Herman 1874, Brunner von Wattenwyl 1882, Redtenbacher 1900, Jacobson and Bianki 1905, Harz 1969) are compared with measurements of a male specimen collected on Vis Is. Shown are body length (from the frons to the tip of abdomen), pronotum length (in dorsal view), length of the visible part of tegmina, hind femur, and ovipositor length (in females). All measurements are in mm. https://binary.pensoft.net/file/414990This dataset is made available under the Open Database License (http://opendatacommons.org/licenses/odbl/1.0/). The Open Database License (ODbL) is a license agreement intended to allow users to freely share, modify, and use this Dataset while maintaining this same freedom for others, provided that the original source and author(s) are credited.Rob Felix, Klaus-Gerhard Heller, Baudewijn Odé, Fran Rebrina, Josip Skejo

Supplementary material 3 *Barbitistes kaltenbachi* bioacoustics and comparative morphology Data type Microsoft Word Document (.docx) Explanation note Table S3.1. Measurements of the amplitudes in the song of *Barbitistes kaltenbachi*; Table S3.2 Measurements of the species-specific time pattern in the song of *Barbitistes kaltenbachi*; Table S3.3 Measurements of the parameters of calling activity of *Barbitistes kaltenbachi*; Table S3.4 Sampling locality data for the specimens used in Fig. 7 (cercus morphology). https://binary.pensoft.net/file/414991This dataset is made available under the Open Database License (http://opendatacommons.org/licenses/odbl/1.0/). The Open Database License (ODbL) is a license agreement intended to allow users to freely share, modify, and use this Dataset while maintaining this same freedom for others, provided that the original source and author(s) are credited.Rob Felix, Klaus-Gerhard Heller, Baudewijn Odé, Fran Rebrina, Josip Skejo
