# Supplementary material for: Island mysteries in the spotlight: Barbitistes kaltenbachi and Rhacocleis buchichii, the only bush-cricket species endemic to Croatia (Orthoptera, Tettigoniidae)
Source: Zookeys. 2020 May 28;936:25–60. doi: 10.3897/zookeys.936.51599 (PMC7272475; doi:10.3897/zookeys.936.51599)
Supplement: Supplementary material 1 — Barbitistes kaltenbachi morphometrics [file zookeys-936-025-s001.docx]

Supplement 1. *Barbitistes kaltenbachi* morphometrics

Supplementary table 1. Morphometrics of Hvar Saw Bush-cricket. Published measurements (Harz 1965, 1969) are compared with the measurements of specimens collected on Vis Is. and Hvar Is. Shown are body length (from the frons to the tip of abdomen), pronotum length (in dorsal view), length of the visible part of tegmina, hind femur, and ovipositor length (in females). All measurements are in mm. [* the tip of abdomen of the other male was eaten by a wasp during preparation]

|  |  | Body length | Pronotum | Elytron | Hind femur | Ovipositor |
| --- | --- | --- | --- | --- | --- | --- |
| Harz (1965, 1969) | ♂ | 20,0 – 22,0 | 5,5 – 6,5 | 4,5 – 5,0 | 18,0 – 21 | n/a |
|  | ♀ | 18,0 – 24,5 | 5,5 – 6,5 | 2,0 – 2,5 | 19,5 – 22 | 11,5 – 13,5 |
| This study, Vis | ♂ | 22,6* | 4,6 – 4,9 | 4,9 – 5,0 | 16,2 – 17,3 | n/a |
|  | ♀ | 26,7 | 6,0 | 2,7 | 20,9 | 14,5 |
| This study, Hvar | ♂ | 24 – 25 | 5,3 – 5,5 | 4,7 – 5,0 | 19 – 22 | n/a |
|  | ♀ | 22 | 6,5 – 6,8 | 2 | 22 – 22,5 | 12 – 13 |
