# Supplementary material for: Island mysteries in the spotlight: Barbitistes kaltenbachi and Rhacocleis buchichii, the only bush-cricket species endemic to Croatia (Orthoptera, Tettigoniidae)
Source: Zookeys. 2020 May 28;936:25–60. doi: 10.3897/zookeys.936.51599 (PMC7272475; doi:10.3897/zookeys.936.51599)
Supplement: Supplementary material 2 — Rhacocleis buchichii morphometrics [file zookeys-936-025-s002.docx]

Supplement 2. *Rhacocleis buchichii* morphometrics

Supplementary table 2. Morphometrics of Lesina Bush-cricket. Published measurements (Herman 1874, Brunner von Wattenwyl 1882, Redtenbacher 1900, Jacobson & Bianki 1905, Harz 1969) are compared with measurements of a male specimen collected on Vis Is. Shown are body length (from the frons to the tip of abdomen), pronotum length (in dorsal view), length of the visible part of tegmina, hind femur, and ovipositor length (in females). All measurements are in mm.

|  |  | Body length | Pronotum | Elytron | Hind femur | Ovipositor |
| --- | --- | --- | --- | --- | --- | --- |
| Herman (1874) | ♂♀ | 22,0 | 7,0 | 2,5 | 22,0 | - |
| Brunner von Wattenwyl (1882) | ♂ | 19,0 | 6,0 | 1,5 | 20,0 | n/a |
|  | ♀ | 22,0 | 8,5 | 0,0 | 26,0 | 18,0 |
| Redtenbacher (1900) | ♂ | 19,0 | - | - | - | n/a |
|  | ♀ | 22,0 | - | - | - | 18,0 |
| Jacobson & Bianki (1905) | ♂ | 19,0 | 6,0 | 1,5 | 20,0 | n/a |
|  | ♀ | 22,0 | 8,5 | 0,0 | 26,0 | 18,0 |
| Harz (1969) | ♂ | 17,5 – 22,0 | 7,0 – 7,5 | 1,5 – 2,0 | 20,5 – 23,0 | n/a |
|  | ♀ | 18,5 – 25,0 | 6,5 – 8,5 | 0,0 | 21,5 – 26,5 | 16,0 – 18,0 |
| This study, Vis | ♂ | 23,3 | 7,1 | 0,8 | 23,1 | n/a |
| This study, Hvar (KGH) | ♂ | 25,0 | 8,0 | 2,0 | 22,5 | n/a |
| This study, Biokovo (KGH) | ♀ | 25,0 | 7,5 | 0 | 24,0 | 17,5 |
