# Supplementary material for: Island mysteries in the spotlight: Barbitistes kaltenbachi and Rhacocleis buchichii, the only bush-cricket species endemic to Croatia (Orthoptera, Tettigoniidae)
Source: Zookeys. 2020 May 28;936:25–60. doi: 10.3897/zookeys.936.51599 (PMC7272475; doi:10.3897/zookeys.936.51599)
Supplement: Supplementary material 3 — Barbitistes kaltenbachi bioacoustics and comparative morphology [file zookeys-936-025-s003.docx]

Supplement 3. *Barbitistes kaltenbachi* bioacoustics and comparative morphology

Supplementary table 3.1. Measurements of the amplitudes in the song of *Barbitistes kaltenbachi*.

|  |  | trigger syllable | | | echeme syllable | | | difference |
| --- | --- | --- | --- | --- | --- | --- | --- | --- |
| Male# file# | T [°C] | relative amplitude | CV [%] | n | relative amplitude | CV [%] | n | [dB] |
| B_1_10 | 18 | 11.0 ± 1.2 | 10.8 | 21 | 4.0 ± 0.9 | 23.6 | 46 | -8.8 |
| B_2_13 | 18 | 15.3 ± 2.1 | 14.0 | 20 | 4.3 ± 1.6 | 38.2 | 60 | -11.1 |
| B_3_20 | 19.5 | 15.9 ± 2.1 | 13.2 | 24 | 2.6 ± 0.6 | 23.7 | 52 | -15.9 |
| B_4_19 | 19.5 | 13.7 ± 1.7 | 12.7 | 21 | 2.3 ± 0.7 | 30.3 | 58 | -15.4 |
| B_5_09 | 22 | 12.3 ± 1.5 | 12.1 | 25 | 6.3 ± 1.1 | 17.6 | 47 | -5.8 |
| S_1_21 | 21.5 | 12.5 ± 2.4 | 19.2 | 24 | 2.8 ± 1.5 | 52.4 | 48 | -12.9 |
| S_2_17 | 19.5 | 14.6 ± 1.8 | 12.2 | 20 | 3.0 ± 1.1 | 35.3 | 63 | -13.7 |

Supplementary table 3.2. Measurements of the species-specific time pattern in the song of *Barbitistes kaltenbachi*.

|  | | verse period | |  | post trigger period | | | | syllable period (echeme) | | | | pre trigger period | | | |
| --- | --- | --- | --- | --- | --- | --- | --- | --- | --- | --- | --- | --- | --- | --- | --- | --- |
| Male_#  _file# | T [°C] | mean ± SD [ms] | CV [%] | n | mean ± SD [ms] | CV [%] | n | mean ± SD [ms] | | CV [%] | n | mean ± SD [ms] | | CV [%] | n |  |
| B_1_10 | 18 | 363.4 ± 12.5 | 3.4 | 21 | 140.9 ± 15.5 | 11.0 | 21 | 50.3 ± 7.2 | | 14.4 | 28 | 158.5 ± 14.8 | | 9.3 | 21 |  |
| B_2_13 | 18 | 382.2 ± 7.4 | 1.9 | 20 | 140.2 ± 4.2 | 3.0 | 20 | 43.8 ± 4.2 | | 9.5 | 40 | 156.7 ± 5.3 | | 3.4 | 20 |  |
| B_3_20 | 19.5 | 320.7 ± 16.0 | 5.0 | 24 | 125.7 ± 11.7 | 9.3 | 24 | 44.4 ± 7.2 | | 16.2 | 28 | 145.1 ± 16.8 | | 11.5 | 24 |  |
| B_4_19 | 19.5 | 335.5 ± 14.3 | 4.3 | 21 | 134.4 ± 7.1 | 5.3 | 21 | 39.0 ± 2.4 | | 6.1 | 37 | 133.4 ± 10.4 | | 7.8 | 21 |  |
| B_5_09 | 22 | 280.6 ± 6.1 | 2.2 | 25 | 121.4 ± 9.0 | 7.4 | 25 | 39.3 ± 3.8 | | 9.6 | 22 | 125.3 ± 9.4 | | 7.5 | 25 |  |
| S_1_21 | 21.5 | 304.8 ± 5.1 | 1.7 | 24 | 126.6 ± 4.2 | 3.3 | 24 | 42.9 ± 2.1 | | 4.9 | 24 | 135.6 ± 4.4 | | 3.2 | 24 |  |
| S_2_17 | 19.5 | 345.4 ± 9.5 | 2.8 | 20 | 126.0 ± 6.1 | 4.8 | 20 | 37.3 ± 2.8 | | 7.4 | 43 | 138.5 ± 9.5 | | 6.9 | 20 |  |

Supplementary table 3.3. Measurements of the parameters of calling activity of *Barbitistes kaltenbachi*.

|  | Duration of series of verses | | | | Interval between series | | duty cycle |
| --- | --- | --- | --- | --- | --- | --- | --- |
| Male_#_file# | mean ± SD  [s] | median  [s] | range  [s] | n | mean ± SD  [s] | n | % |
| B_1_18 | 15.9 ± 10.3 | 15.8 | 1.7-31.9 | 10 | 2.4 ± 2.0 | 9 | 87.9 |
| B_2_14 | 13.2 ± 5.1 | 11.9 | 4.9-23.1 | 17 | 6.5 ± 2.6 | 17 | 67.1 |
| B_3_17 | 8.0 ± 1.8 | 7.4 | 6.3-12.1 | 10 | 8.8 ± 1.7 | 10 | 47.7 |
| B_4_14 | 13.0 ± 5.6 | 12.3 | 3.7-21.3 | 10 | 19.6 ± 20.0 | 10 | 39.9 |
| B_5_09 | 8.5 ± 3.3 | 8.5 | 6.2-10.8 | 2 | 19.0 | 1 | 47.2 |
| S_1_18 | 7.4 ± 4.1 | 8.0 | 1.3-15.8 | 15 | 5.1 ± 2.9 | 14 | 61.0 |
| S_2_17 | 12.5 ± 3.5 | 12.7 | 3.8-19.5 | 12 | 3.1 ± 0.7 | 11 | 81.7 |

Supplementary table 3.4. Sampling locality data for the specimens used in Fig. 7 (cercus morphology)

| CH0860 | *Barbitistes alpinus* Fruhstorfer, 1920 = obtusus Targioni-Tozzetti, 1881 | ITALY: Trentino, Mt. Pasubio (45°48’N, 11°11’E), 29 ix 1980, leg. D.v.Helversen |
| --- | --- | --- |
| CH0867 | *Barbitistes constrictus* Brunner von Wattenwyl, 1878 | GERMANY: Bayern, Erlangen (49°35’N, 11°0’E), 14 vi 1982, leg. W. Edrich |
| CH0863 | *Barbitistes fischeri* (Yersin, 1854) | FRANCE: Al.-Haute-Prov., Col de Fontbelle bei Digne (44°13’N, 6°9’E), 1160 m, 2 ix 1980, leg. Heller & Volleth |
| CH6735 | *Barbitistes kaltenbachi* Harz, 1965 | CROATIA: Dalmatia, Island of Hvar, Bogomolje (Likova Glava), 30 v 2006, leg. M. & K.-G. Heller |
| CH8388 | *Barbitistes ocskayi* Charpentier, 1850 | MONTENEGRO: Medun (42°28’N, 19°22’E), 500 m, 6 vi 2017, leg. Martina Heller |
| CH3131 | *Barbitistes serricauda* (Fabricius, 1794) | SWITZERLAND: Wallis, Salvan; Val d’En Haut (46°7’N, 7°0’E), 1450 m, 25 vii 1994, leg. K.-G.Heller |
| CH6099 | *Barbitistes vicetinus* Galvagni & Fontana, 1993 | ITALY: Veneto (VI), Propaggini dei M. Lessini. Isola Vicentina, Torreselle Cima (45°36’N, 11°24’E), 12 vi 1999, leg. P. Fontana |
| CH8389 | *Barbitistes yersini* Brunner von Wattenwyl, 1875 | MONTENEGRO: Korita (42°29’N, 19°33’E), 1350 m, 6 vi 2017, leg. Martina & Klaus-Gerhard Heller |
